# Transcriptional Activation of Transposable Element (TE)-Associated Genes Is Frequently Associated with Altered Promoter Methylation in Placenta and Melanoma

**DOI:** 10.3390/ijms27135827

**Published:** 2026-06-27

**Authors:** Chiemi F. Lynch-Sutherland, Lorissa I. McDougall, Peter A. Stockwell, Aniruddha Chatterjee, Teena K. J. B. Gamage, Joanna L. James, Euan J. Rodger, Robert J. Weeks, Jackie L. Ludgate, Erin C. Macaulay, Michael R. Eccles

**Affiliations:** 1 Department of Pathology and Molecular Medicine, Dunedin School of Medicine, University of Otago, P.O. Box 56, Dunedin 9054, New Zealand; clynch-sutherland@cmri.org.au (C.F.L.-S.); lorissa.mcdougall@gmail.com (L.I.M.); peter.stockwell@otago.ac.nz (P.A.S.); aniruddha.chatterjee@otago.ac.nz (A.C.); euan.rodger@otago.ac.nz (E.J.R.); robjweeks@gmail.com (R.J.W.); jackie.ludgate@otago.ac.nz (J.L.L.); erin.macaulay@otago.ac.nz (E.C.M.); 2 Children’s Medical Research Institute, Westmead, NSW 2145, Australia; 3 Kids Neuroscience Centre, Kids Research, The Children’s Hospital at Westmead, Westmead, NSW 2145, Australia; 4 Maurice Wilkins Centre for Molecular Biodiscovery, The University of Auckland, Level 2, 3A Symonds Street, Private Bag 92019, Auckland 1010, New Zealand; 5 Department of Physiology, Faculty of Medical and Health Sciences, The University of Auckland, Auckland 1010, New Zealand; t.gamage@auckland.ac.nz; 6 Auckland Bioengineering Institute, The University of Auckland, Auckland 1010, New Zealand; 7 Department of Obstetrics, Gynaecology, and Reproductive Sciences, Faculty of Medical and Health Sciences, The University of Auckland, Auckland 1010, New Zealand; j.james@auckland.ac.nz

**Keywords:** transposable elements, endogenous retroviruses, placental-enriched, human-embryonic-stem-cell-enriched, melanoma, TE-associated transcripts

## Abstract

Transposable elements (TEs) play important roles during development and disease, including through transcriptional activation of TE-associated genes during early human development. Moreover, based on the functional and epigenetic similarities between early development and cancer, TE-associated genes contribute not only to early human development, but frequently contribute to cancer progression. In this study, we hypothesised that recruitment of TE-associated genes during cancer onset occurs through epigenetic regulatory processes, especially involving DNA hypomethylation accompanied by transcriptional upregulation of early developmental pathways, such that, when reactivated inappropriately in later life, they may drive malignancy. It is unknown, however, to what extent DNA methylation changes are critically involved in the transcriptional activation of TE-associated genes. Accordingly, to investigate this we used the RepExpress tool to identify developmentally regulated TE-associated genes in placenta and human embryonic stem cells (hESCs), which we then investigated by targeted deep bisulfite sequencing (TDBS) to determine the methylation status of the identified TE-associated genes in placenta, somatic tissues, and melanoma cell lines. Outcomes suggest that DNA methylation may be one of the regulatory factors underscoring transcriptional activation of TE-associated genes, but that methylation is not necessarily the sole factor involved in regulating the transcriptional activation of TE-associated genes during malignant transformation.

## 1. Introduction

Transposable element (TE) activation is a well-established feature of cancer, and is thought to be accompanied by loss of DNA methylation [1,2,3,4,5,6,7]. Early work focused on transposition-mediated mutagenesis as a driver of tumorigenesis [8,9,10]. However, it is now clear that TE activation contributes to cancer through additional mechanisms, including modulation of immune responses via endogenous retrovirus (ERV) expression and the co-option of TE sequences to regulate oncogene expression (onco-exaptation) [11,12]. Furthermore, TE-associated genes can function as oncogenes [13,14]. However, despite establishing that TE expression is linked to oncogenesis, many uncertainties remain, such as whether localised gene-specific demethylation is frequently associated with transcriptional activation of TE-associated genes in various types of cancer. Additionally, with respect to the functional implications of TE-associated gene expression in disease processes such as cancer, whether TE-associated genes are potentially involved in promoting early developmental processes in cancer is not clear.

Activation of developmental genes is frequently associated with tumour progression [15,16,17,18,19]. Cancers recapitulate some aspects of early development, such as invasion, proliferation, and angiogenesis [20,21,22]. Further, loss of cellular identity is a feature of advanced cancer stages, and certain cancers harbour cells with properties resembling stem cells, conferring aggressive features with increased oncogenic potential [23]. Given the known roles of TE-associated genes in regulating developmental processes, along with evidence for transcriptional activation of TE-associated genes in cancer [15,16], it is possible that reactivation of TE-associated genes may be involved in early developmental-like features in cancers, promoting malignancy and tumour progression. If so, then does transcriptional activation of TE-associated genes in cancer result from, and/or could it act as a driver of, epigenetic reprogramming of cancer cells? In this regard, we have previously shown that placental TE-associated genes lacking DNA methylation are expressed in melanoma cell lines [24].

It is widely assumed that reduced DNA methylation underlies the activation of TE-associated genes in pluripotent tissues and cancer [3,25,26,27,28]. However, this assumption has rarely been tested systematically across multiple loci. Rather than focusing on individual genes, a broader approach is needed to determine whether DNA methylation is consistently associated with TE-associated gene activation in placenta, somatic tissues, in early developmental tissues such as in hESCs, or in cancer.

This study investigates whether altered DNA methylation is consistently associated with transcriptional activation of TE-associated genes in pluripotent and malignant tissues. Specifically, we assess (i) the extent to which placental- and human-embryonic-stem-cell (hESC)-enriched TE-associated genes are reactivated in melanoma, and (ii) whether DNA methylation correlates with their expression across tissue types.

We identified placental- and hESC-enriched TE-associated genes using RepExpress [29], and examined their expression and DNA methylation across placenta, hESCs, somatic tissues, and melanoma cell lines. While TE-associated genes were frequently upregulated alongside altered promoter methylation, this relationship was not universal, indicating that DNA methylation is not the sole regulator of their activation.

## 2. Results

### 2.1. Placental- and hESC-Enriched TE-Associated Genes, Including Both Long Non-Coding RNAs and Protein-Coding Genes, Are Often Highly Expressed in Placenta and Melanoma Cells, but Expressed at Low Levels in Somatic Tissues

To identify placental- and hESC-enriched TE-associated genes we used the RepExpress [29] TE identification tool and RNA-Sequencing data performed on total RNA (a summary of the workflow is provided in Supplementary Material, Figure S1). In total, 21 placental-enriched and 28 hESC-enriched TE-associated lncRNAs, together with 28 and 45 corresponding protein-coding genes, exhibited at least two-fold higher expression in melanoma cell lines relative to somatic tissues and were expressed in both placenta and melanoma.

**Figure 1:**
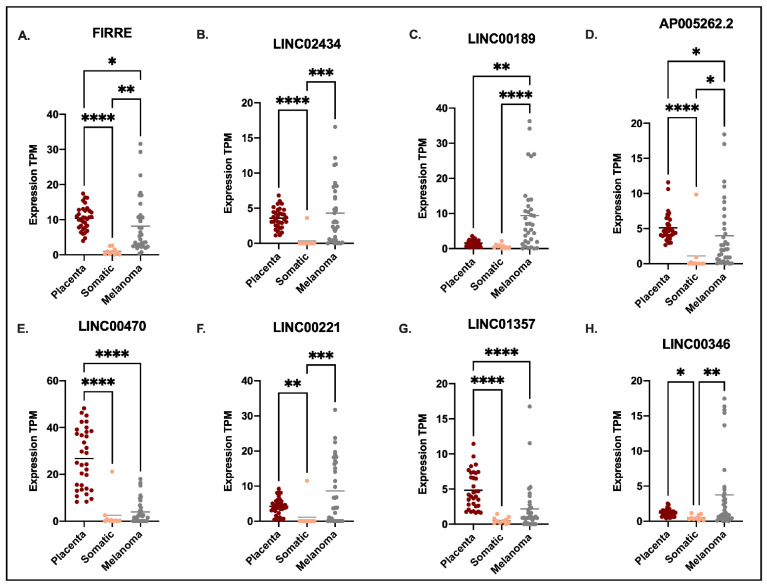
Expression of placental-enriched lncRNAs in placental tissues, somatic tissues, and melanoma cell lines. (**A**) *FIRRE*. (**B**) *LINC02434*. (**C**) *LINC00189*. (**D**) *AP005262.2*. (**E**) *LINC00470*. (**F**) *LINC00221*. (**G**) *LINC01357*. (**H**) *LINC00348*. Placenta n = 33 (17 first trimester and 16 term), somatic n = 8 (brain, heart, liver, kidney, lung, ovary, testis, melanocyte), melanoma cell lines n = 34. Asterisks denote statistical significance; **** = *p* value < 0.0001, *** = *p* value 0.0001–0.001, ** = *p* value 0.001–0.01, * = *p* value 0.01–0.05; Kruskal–Wallis test.

**Figure 2:**
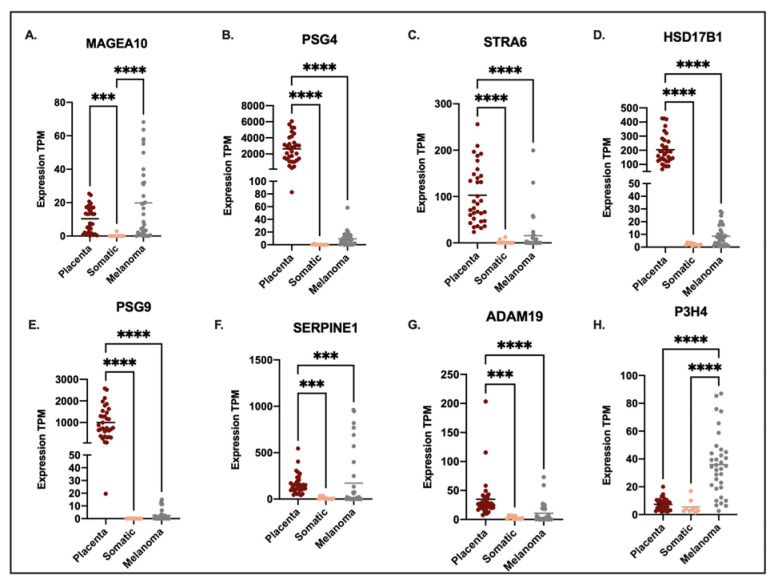
Expression of placental-enriched protein-coding genes in placental tissues, somatic tissues, and melanoma cell lines. (**A**) *MAGEA10*. (**B**) *PSG4*. (C) *STRA6*. (**D**) *HSD17B1*. (**E**) *PSG9*. (**F**) *SERPINE1*. (**G**) *ADAM19*. (**H**) *P3H4*. See Figure 1 legend for sample numbers and statistics definitions.

**Figure 3:**
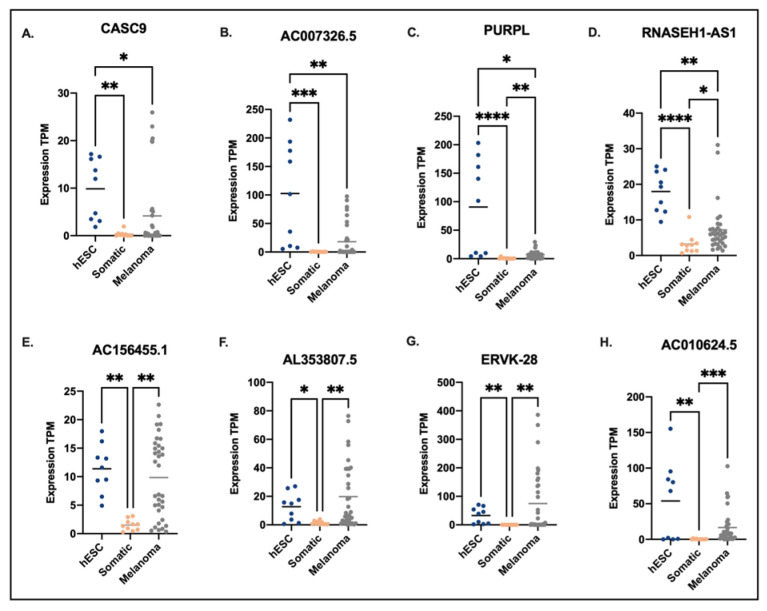
Expression of hESC-enriched lncRNAs in hESC, somatic tissues, and melanoma cell lines. (**A**) *CASC9*. (**B**) *AC007326.5*. (**C**) *PURPL*. (**D**) *RNASEH1-AS1*. (**E**) *AC156455.1*. (**F**) *AL353807.5*. (**G**) *ERVK-28*. (**H**) *AC010624.5*. hESC n = 8, somatic n = 8 (brain, heart, liver, kidney, lung, ovary, testis, melanocyte) melanoma cell lines n = 34. Asterisks denote statistical significance; **** = *p* value < 0.0001, *** = *p* value 0.0001–0.001, ** = *p* value 0.001–0.01, * = *p* value 0.01–0.05; Kruskal–Wallis test.

**Figure 4:**
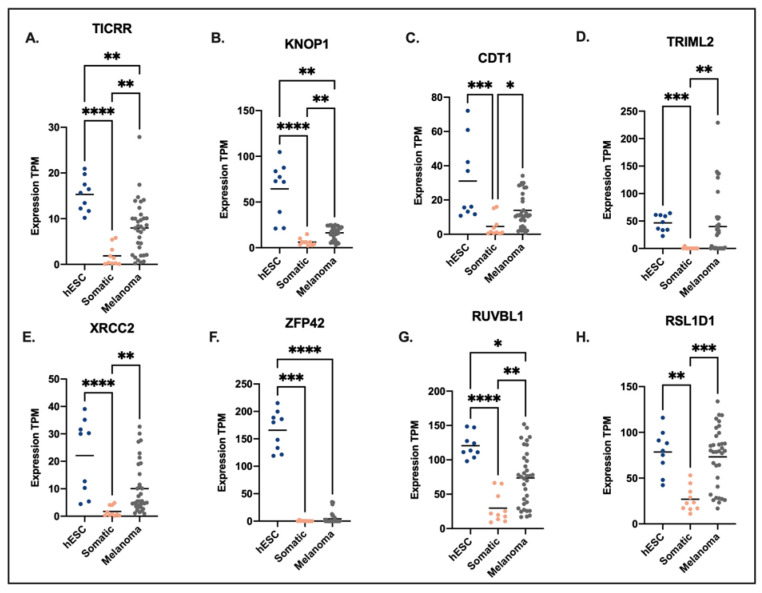
Expression of hESC-enriched protein-coding genes in hESC, somatic tissues, and melanoma cell lines. (**A**) *TICRR*. (**B**) *KNOP1*. (**C**) *CDT1*. (**D**) *TRIML2*. (**E**) *XRCC2*. (**F**) *ZFP42*. (**G**) *RUVBL1*. (**H**) *RSL1D1*. See Figure 3 legend for sample numbers and statistics definitions.

Based on their expression levels (for instance, loci showing significant differential expression between placental and/or melanoma and somatic tissue), representative TE-associated placental-enriched and hESC-enriched long non-coding RNAs (lncRNAs), as well as protein-coding genes, were selected (including *FIRRE*, *LINC02434*, *LINC00189*, *AP005262.2*, *LINC00470*, *LINC00221*, *LINC01357*, *LINC00346*, *MAGEA10*, *PSG4*, *STRA6*, *HSD17B1*, *PSG9*, *SERPINE1*, *ADAM19*, *P3H4*, *CASC9*, *AC007326.5*, *PURPL*, *RNASEH1-AS1*, *AC156455.1*, *AL353807.5*, *ERVK-28*, *AC010624.5*, *TICRR*, *KNOP1*, *CDT1*, *TRIML2*, *XRCC2*, *ZFP42*, *RUVBL1*, *RSL1D1*, shown in Figure 1, Figure 2, Figure 3 and Figure 4) to show expression patterns in placental villous tissue (first trimester and term) or hESCs, alongside somatic tissues (brain, heart, kidney, liver, lung, ovary, testis, and melanocyte) and melanoma cell lines. With respect to eight selected lncRNAs from placental-enriched TE-associated genes, and eight selected protein-coding genes, in many cases transcription was significantly upregulated in placental villous tissue and melanoma cell lines when compared to healthy somatic tissues (Figure 1 and Figure 2), albeit there were fewer TE-associated protein-coding genes in this regard than lncRNAs.

Similarly, with respect to eight selected hESC-enriched TE-associated lncRNAs, and eight selected protein-coding genes, many transcripts were significantly upregulated in hESC and melanoma cell lines when compared to healthy somatic tissues (Figure 3 and Figure 4).

### 2.2. A Subset of Placental-Enriched and hESC-Enriched Transposable-Element-Derived Genes Exhibit an Inverse Relationship Between Transcriptional Activity and Promoter DNA Methylation in Placenta and/or Melanoma Cell Lines and Somatic Tissues

We next sought to determine whether the methylation status of promoter regions of TE-associated placental-enriched genes and TE-associated hESC-enriched genes were altered in concert (either directly related or inversely related) with gene expression changes in melanoma cell lines, somatic tissues (blood and melanocyte), and placental villous tissues (first trimester and term). Targeted deep bisulfite sequencing (TDBS) was therefore used to analyse DNA methylation levels in comparison to gene expression levels (generated by RNA-Seq data). Including several of the 32 placental- and hESC-enriched protein-coding and lnc-RNA transcripts analysed above, plus additional TE-associated transcripts, a subset of placental-enriched TE-associated transcripts (four lncRNAs, one protein-coding, and one pseudogene) and hESC-enriched TE-associated genes (three lncRNAs and one protein-coding) was selected based on the placental, somatic tissue, hESC, and melanoma cell line RNA-Seq data. For this analysis, transcripts showing the highest expression in placental tissue, hESC, and melanoma cell lines, and the lowest expression in somatic tissues were specifically prioritised, as well as genes that had TEs within transcription start sites (TSSs), which could potentially suggest a key role for TEs in regulating the expression of these genes. The selected placental-enriched and hESC-enriched TE-associated loci for comparing levels of transcription with DNA methylation were *LINC00470*, *AP005262.2*, *PLAC4*, *HSD17B1*, *LINC00221*, *AC073262.3*, *LINC02575*, and *LINC00698*. More information about these loci is provided in the Supplementary Material, Table S1. Primers for TDBS were designed to cover the maximum number of CpG sites within amplicons. Due to a lack of DNA from hESCs, methylation of TE-associated genes in DNA from hESC cells was not determined.

**Figure 5:**
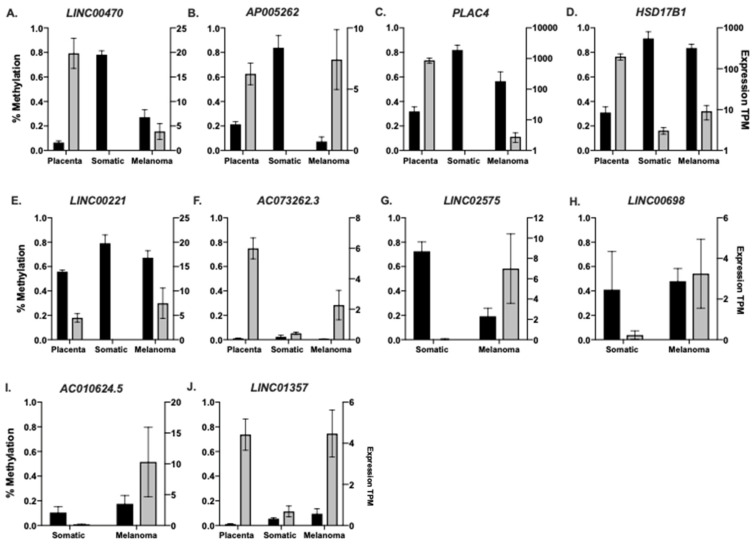
Comparison of gene expression levels versus promoter DNA methylation levels of candidate placental-enriched and hESC-enriched TE-associated genes in placenta and/or somatic tissues and melanoma cell lines. (**A**) *LINC00470*. (**B**) *AP005262.2*. (**C**) *PLAC4*. (**D**) *HSD17B1*. (**E**) *LINC00221*. (**F**) *AC073262.3*. (**G**) *LINC02575*. (**H**) *LINC00698* (**I**) *AC010624.5* (**J**) *LINC01357*. Methylation levels of the promoter regions were determined by TDBS, and expression was quantified by RNA-Sequencing. Mean promoter methylation (black bar and left Y-axis) and expression levels in TPM (grey bar and right Y-axis) of candidate genes in different cell/tissue types (error bars represent SEM). Placenta n = 15, somatic n = 3, melanoma cell line n = 10 (TDBS); placenta n = 33, somatic n = 8, and melanoma cell line n = 34 (RNA-Seq).

Inverse relationships were suggested by this analysis from only a subset of the TE-associated genes (*LINC00470*, *AP005262.2*, *PLAC4*, *HSD17B1*, *LINC00221*, *LINC002575*) when TE-associated gene methylation data were compared to RNA-Sequencing expression data for each of placenta, somatic tissues, and melanoma cell lines (Figure 5). Several TE-associated genes (*AC073262.3*, *LINC00698*, *AC010624.5*, *LINC01357*) showed inconsistent relationships between DNA methylation and expression across cell/tissue types (Figure 5).

#### DNA Methylation Analysis in a Select Group of Placental-Enriched and hESC-Enriched TE-Associated Genes Demonstrate That Methylation of a Cluster of CpG Sites Within Promoter Amplicon Regions Are Inversely Correlated with Transcriptional Activity in Placenta and/or Melanoma Cell Lines

**Figure 6:**
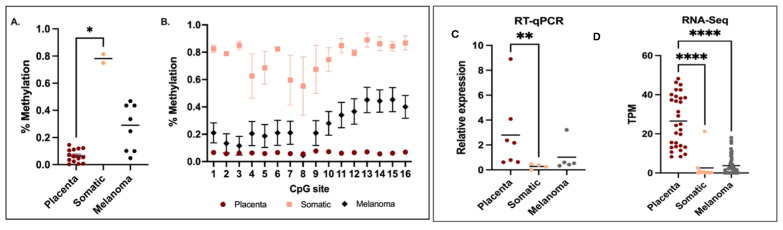
DNA methylation analysis across a LINC00470 amplicon, and quantification of expression of LINC00470 by RT-qPCR and RNA-Sequencing. (**A**) Mean CpG methylation for the LINC00470 amplicon (* = *p* value = 0.0177; Kruskal–Wallis test). (**B**) Methylation of individual CpG sites within the LINC00470 amplicon. Placenta n = 15, somatic n = 2, melanoma cell line n = 10. (**C**) Expression of LINC00470 in placenta, somatic tissue, and melanoma as quantified by RT-qPCR (placenta n = 7, somatic n = 5, melanoma n = 5). (**D**) Expression of LINC00470 in placenta, somatic tissue, and melanoma RNA-Seq datasets (placenta n = 33, somatic n = 8, melanoma n = 34). Asterisks denote statistical significance; **** = *p* value < 0.0001, ** = *p* value 0.001–0.01, * = *p* value 0.01–0.05; Kruskal–Wallis test.

*LINC00470* is a placental-enriched lncRNA, which contains multiple TEs within its transcript sequence, and whose TSS falls within a short-interspersed nuclear element (SINE). Investigation of an amplicon containing 16 CpG sites immediately upstream of the TSS of *LINC00470*, to quantify promoter methylation, showed lower *LINC00470* promoter CpG mean methylation levels in the placental tissue and melanoma cell lines compared to healthy somatic tissue (*p* = 0.0177* for placental tissue versus somatic tissue), although mean methylation differences between melanoma cell lines and somatic tissues or placental tissue were not significant (Figure 6A). The first nine individual CpG sites in the melanoma cell lines showed methylation levels approaching those of placental tissue, as compared to the last seven CpG sites, which showed increased CpG methylation levels (Figure 6B).

Assessment of *LINC00470* expression using RT-qPCR was carried out to validate the RNA-Sequencing data, which showed that there was significant upregulation in placenta compared to somatic tissues (Figure 6C,D). No significant upregulation was observed for *LINC00470* expression as determined by RT-qPCR or RNA-Sequencing of melanoma cell lines.

**Figure 7:**
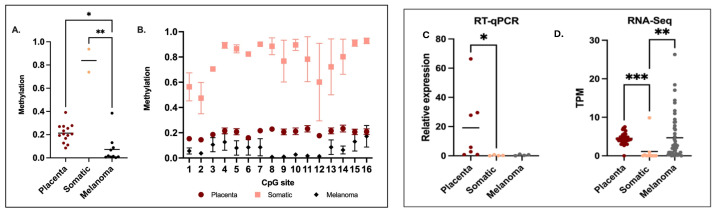
DNA methylation analysis across an AP005262.2 amplicon, and quantification of expression of AP005262.2 by RT-qPCR and RNA-Sequencing. (**A**) Mean CpG methylation for the AP005262.2 amplicon (* = *p* value = 0.0124, ** = *p* value 0.006; Kruskal–Wallis test). (**B**) Methylation of each CpG within the Ap005262 amplicon. Placental tissue n = 15, somatic tissue n = 2, melanoma cell line n = 10. (**C**) Expression of AP005262.2 in placenta, somatic tissue, and melanoma as quantified by RT-qPCR. See Figure 6 legend for sample numbers and statistics definitions. (**D**) Expression of AP005262.2 in placenta, somatic tissue, and melanoma RNA-Seq datasets (placenta n = 33, somatic tissue n = 8, melanoma n = 34). Asterisks denote statistical significance; *** = *p* value 0.0001–0.001, ** = *p* value 0.001–0.01, * = *p* value 0.01–0.05; Kruskal–Wallis test.

*AP005262.2* is a placental-enriched lncRNA, with its TSS occurring within a SINE contained within the 247 bp promoter amplicon region that includes 16 CpG sites. Lower *AP005262.2* promoter CpG mean methylation levels were observed in the placental tissue and melanoma cell lines compared to healthy somatic tissue (Figure 7A; *p* = 0.0124*, *p* = 0.006**). Each individual CpG site within the amplicon showed similar methylation patterns as observed for the mean promoter methylation, although interindividual variation in DNA methylation measured at distinct CpG sites for the two somatic tissue samples was high (Figure 7B).

Assessment of *AP005262.2* expression using RT-qPCR to validate the RNA-Sequencing data showed that there was significant upregulation in placental tissue compared to somatic tissues (Figure 7C,D). Very little upregulation was observed in the RT-qPCR of melanoma cell lines, although only four melanoma cell lines were used for the RT-qPCR. A small proportion of the melanoma cell lines showed very low transcription of *AP005262.2* by RNA-Sequencing.

**Figure 8:**
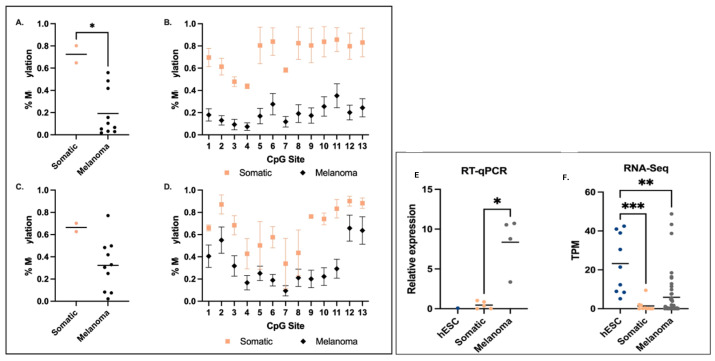
DNA methylation analysis across LINC02575 amplicons, and quantification of expression of LINC02575 by RT-qPCR and RNA-Sequencing. (**A**) Mean methylation levels of promoter region (amplicon one) within LINC02575 (* = *p* value = 0.0303; Mann–Whitney test). (**B**) Mean methylation levels for each CpG site within amplicon one of the LINC02575 promoter. (**C**) Mean methylation levels of promoter region (amplicon two) within the LINC02575 promoter (*p* value = 0.1212; Mann–Whitney test). (**D**) Methylation levels of each CpG within the LINC02575 amplicon two (error bars represent SEM). Somatic tissue types n = 2, melanoma cell line n = 10. (**E**) Expression of LINC02575 in hESCs, somatic tissue, and melanoma, as quantified by RT-qPCR (hESC n = 1, somatic tissue types n = 5, melanoma n = 5). (**F**) Expression of LINC02575 in hESCs, somatic tissue types, and melanoma RNA-Seq datasets (hESC n = 8, somatic tissue n = 8, melanoma n = 34). Asterisks denote statistical significance; *** = *p* value 0.0001–0.001, ** = *p* value 0.001–0.01, * = *p* value 0.01–0.05; Kruskal–Wallis test.

*LINC02575* is an hESC-enriched lncRNA, whose entire transcript sequence is derived from long terminal repeat (LTR) elements. Primers were designed to cover two regions within the *LINC02575* promoter; firstly, a 191 bp fragment containing 13 CpG sites, and secondly, a 213 bp amplicon further upstream from the TSS, which also contained 13 CpG sites. Mean promoter methylation levels for the two amplicons of *LINC02575* were lower in melanoma cell lines compared to healthy somatic tissues, although this difference was only significant for the first amplicon, which was located closer to the transcriptional start site of *LINC02575* (Figure 8A,C). Methylation levels for each CpG site for both amplicons showed similar patterns to those of the mean promoter methylation (Figure 8B,D), although the second amplicon displayed more interindividual variation in melanoma cell lines.

Assessment of *LINC02575* expression using RT-qPCR to validate the RNA-Sequencing data showed that there was significant upregulation in melanoma cell lines compared to somatic tissues (Figure 8E,F). However, no upregulation was observed in the RT-qPCR of hESC cells, although only one hESC cell line was used for this RT-qPCR analysis.

Lastly, we carried out an analysis of CpG methylation using reduced representation bisulfite sequencing (RRBS) of normal melanocyte cell lines as well as in melanoma cell lines, as analysed above by TDBS, which agreed well with TDBS data (see Supplementary Figure S2).

## 3. Discussion

A key finding of this study is that TE-associated genes are frequently, but not consistently, transcriptionally upregulated in association with altered promoter methylation in placenta and melanoma, relative to somatic tissues. This supports a role for DNA methylation in regulating TE-associated gene expression, but these results indicate that methylation is not the sole determinant of TE-associated gene expression.

Regarding the selection of TE-associated genes included in this study, in many cases the TE-associated genes were newly identified (using RepExpress), placenta-enriched, and hESC-enriched TE-associated lncRNAs, as well as protein-coding TE-associated genes. Many of the transcripts were significantly upregulated in placental tissues, hESCs, and melanoma cell lines when compared to healthy somatic tissues, which suggests that they may have roles specific to early human development, and these putative roles could potentially be related to their becoming reactivated in melanoma. In some instances, TE-associated genes were significantly upregulated in specific subgroups of melanoma cell lines (see Supplementary Figures S3–S10), as well as in multiple other cancer types (for example, see Supplementary Figures S11–S13). Relative expression levels of TE-associated genes in RNA-Sequencing data were subsequently validated using RT-qPCR (also see Supplementary Figures S14–S16).

Following expression analysis, TDBS was then used as the primary method for DNA methylation anaysis. Due to the repetitive nature of DNA (as represented by TE-associated gene sequences), regions within TE-associated genes are frequently problematic for standard sequencing pipelines, hence the need for TDBS. The TDBS analysis revealed that putative TE-associated gene promoters frequently showed a range of different methylation patterns across the tissues and cell lines investigated. In the somatic tissues surveyed, numerous TE-associated gene promoters were hypermethylated (70–90%), and yet many other TE-associated gene promoters were hypomethylated. In melanoma cell lines, a range of methylation levels were observed in different cell lines, with some cell lines showing mean methylation levels below 30% for many TE-associated genes. Overall, TE-associated gene hypomethylation at canonical promoter regions was more common in melanoma cell lines than in somatic tissues, while in contrast, TE-associated gene promoter hypermethylation was more common in somatic tissues; however, some TE-associated genes in melanoma cell lines exhibited promoter hypermethylation.

Investigation of individual CpG methylation in three selected TE-associated gene promoters demonstrated that the same general trend occurs in individual CpGs as the mean methylation for the different tissue types (additional CpG methylation profiles in TE-associated gene promoters are shown in Supplementary Figures S15–S23). Interestingly, using RRBS methylation analysis of multiple CpG sites in the TE-associated gene, *ADAM19*, it was evident that results from three different normal melanocyte cell lines showed promoter hypomethylation, yet there was no *ADAM19* RNA expression observed in somatic tissues, including in melanocyte cell lines, although variable levels of methylation and expression of *ADAM19* were observed in melanoma cell lines (see Supplementary Figure S2, and Supplementary Table S12, compared to Figure 2).

It was previously reported that hypomethylation of TE-associated genes in melanoma occurs in association with the metastatic capacity of primary melanomas [30]. However, the results from this study suggest that hypomethylation alterations involving TE-associated genes are not consistently associated with transcriptional upregulation of TE-associated genes in placenta and melanoma [19,31,32,33,34,35,36]. In support of this, *AC073262.3*, *AC010624.5*, and *LINC01357* (see Supplementary Figures S20, S22 and S23) had low levels of methylation in all tissue and cell types, despite significant differences in their expression across different tissue and cell types, indicating that other epigenetic mechanisms must regulate their expression. Additionally, *LINC00698* (see Supplementary Figure S21) had intermediate levels of methylation that were comparable in both somatic tissue and melanoma cell lines, despite *LINC00698* showing significant differences in expression.

With respect to the TE-associated genes, *PLAC4*, *HSD17B1*, *LINC00470*, and *AP005262.2* (out of interest, the TSS SINE of AP005262.2 is conserved only within some higher-order primate species, but not in other placental mammals), there were low levels of methylation in the placenta that corresponded to high levels of expression, whilst the opposite was observed in somatic tissues. In this regard, *PLAC4* and *HSD17B1* showed an inverse relationship between promoter methylation and expression in placenta and somatic tissue, but this was not true in melanoma cell lines. Further, regarding *LINC00470* and *AP005262.2*, the melanoma cell lines showed intermediate levels of mean promoter methylation and these genes were also expressed at intermediate levels in melanoma. During a preliminary analysis, we identified several of the TE-associated transcripts identified in this study in pre-treatment DNA methylome and transcriptome profiles, which correlated with melanoma response to anti-PD1 immunotherapy [17]. Although *LINC02575* has not been characterised functionally in hESCs, a very recent study identified *LINC02575* as being prognostic in laryngeal squamous cell carcinoma (LSCC) [37]. This study revealed that *LINC02575* is capable of promoting proliferation, migration, and invasion in LSCC cell lines. *LINC02575* also demonstrated an inverse relationship between promoter methylation and expression, with promoter methylation being high in somatic tissues and low in melanoma cell lines, with the inverse being true regarding expression levels. In contrast to DNA methylation, epigenetic alterations involving chromatin-associated changes have been linked with transcriptional activation of TE-associated genes in hESC cell lines and in cancer [38,39].

It has been proposed that transcription, together with methylation patterns associated with TE-associated sequences, has played a major role in the evolution of human-specific (as well as primate-specific) gene regulation [40]. However, whether DNA hypomethylation alterations determine the subsequent transcriptional activation of TE-associated genes appears complex. In this regard, previous studies have often focused on only one or several retrospectively discovered TE-associated genes [41,42,43,44], and relatively few studies have employed genome-wide TE-associated gene discovery, together with methylation and transcription analysis techniques [45].

Recently, an evolutionary model was proposed in which vertebrate TEs are indirectly responsible for the existence of CpG islands, as well as for the formation of regulatory elements such as TSSs and enhancers that can potentially utilise dynamic DNA methylation for gene control [46]. It has also been suggested that DNA hypomethylation in O-GlcNAc transferase (OGT)-deficient cells is accompanied by derepression of TEs predominantly located in heterochromatin [47]. OGT is suggested to protect the genome against TET-mediated DNA demethylation and loss of heterochromatin integrity, preventing aberrant increases in TE expression as noted in cancer, autoimmune-inflammatory diseases, cellular senescence, and ageing [47]. However, the role of DNA methylation in cancer is complex because in many cancers global DNA hypomethylation is accompanied by hypermethylation of tumour suppressor gene promoters, with associated tumour suppressor gene silencing. A wide variety of cancers display both DNA hypomethylation and hypermethylation, and either of these types of changes can be significantly associated with tumour progression [48]. Moreover, comparisons between methylation levels of the LTR-derived gene promoters and six random HERV-E LTRs in human placenta showed that the former display significantly lower methylation levels than random LTRs [49]. Therefore, other factors are also involved in regulating TE-associated genes, as is evident in mice harbouring a whole human chromosome 21 in the mouse nucleus, whereby hundreds of locations on the human chromosome 21 were newly associated with activating histone modifications in both somatic and germline tissues, and which influenced gene expression of nearby transcripts [50].

In conclusion, we found that TE-associated genes in many cases (although not universally) were transcriptionally upregulated in concert with altered promoter methylation in placental tissues, hESCs, somatic tissues, and melanoma cell lines. These results suggest that, since methylation changes were not consistently associated in many of the TE-associated genes exhibiting transcriptional upregulation in melanoma cell lines, DNA methylation alterations constitute just one of several factors regulating transcriptional activation of TE-associated genes during malignant transformation.

## 4. Materials and Methods

### 4.1. Tissue and Cell Line Samples Used for RNA-Sequencing Analysis

The study utilised two cohorts of first-trimester placental villous tissue samples from healthy pregnancies in Auckland and Dunedin (New Zealand). Auckland first-trimester placental samples (7–12.5 weeks’ gestation) were collected at Auckland City Hospital, and Dunedin first-trimester placental samples (5–9 weeks’ gestation) were collected at Dunedin Hospital, both following informed consent (Supplementary Table S2). All placental samples were processed within four hours, as previously described [51,52,53]. Publicly available data generated from term placental samples were obtained from the sequence read archive (SRA-https://www.ncbi.nlm.nih.gov/sra, accessed on 14 June 2026) (Supplementary Table S3). One commercially available human embryonic stem cell line was obtained from Australian Biosearch (Wangarra, WA, Australia), and publicly available stem cell RNA-Seq data were curated for analysis (Supplementary Table S4). Commercially available RNA was purchased from AMSBIO (Cambridge, UK) for RNA-Sequencing from seven healthy somatic tissues (Supplementary Table S4). Melanoma and melanocyte cell lines were used for RNA-Sequencing, as described [17] (also see Supplementary Table S5).

### 4.2. Identification and Analysis of TE-Associated Genes from RNA-Sequencing Data

RNA was extracted for RNA-Sequencing analysis as described previously [17]. During preliminary analyses for RNA-Sequencing it was observed that a significant proportion of TEs were upregulated in placental tissue/hESCs in comparison to mean expression levels in healthy somatic tissues. Placental tissue/hESC-enriched genes were classified by an average expression count of more than 80, and a fold change of more than 3 in placental tissue/hESCs when compared to the average expression of the eight somatic tissues.

Bioinformatic analysis of RNA-Sequencing data (also involving RepExpress) was carried out as described previously [17,29]. All RNA-Seq libraries were ribodepleted, except one of the hESC datasets, two of the three melanocyte samples, and 12 of the melanoma cell lines, which were poly A-selected (see Supplementary Tables S2–S5). Significant variations were noted between sequencing depths (notably, the melanoma datasets had substantially lower depth than the placental), which potentially impacted the results through reducing the number of TE-associated genes identified to be reactivated in melanoma.

### 4.3. Validation of RNA-Sequencing Data Using RT-qPCR

Briefly, for RT-qPCR analysis, RT-qPCR primers (from IDT, Singapore) were designed for candidate genes (Supplementary Tables S6 and S7). Four genes were placental-enriched TE-associated genes, and two were hESC-enriched. All selected genes were also upregulated in melanoma in comparison to healthy somatic tissue. Six first-trimester placental samples, five term, one hESC, five somatic tissues, and six melanoma cell lines were selected based on the availability of RNA-Seq data and those used for the targeted methylation assays (Supplementary Table S8) (expressed in hESC and placenta).

### 4.4. Targeted Deep Bisulfite Sequencing (TDBS) Assays

Genomic DNA was extracted for TDBS assays, as described previously [51]. Briefly, four TDBS PCR primers (from IDT, Singapore) were designed (Supplementary Tables S9 and S10) for the promoter regions of 10 candidate TE-associated genes using the MethPrimer tool. The first exon along with ~250 bp upstream was provided as input to the tool. Optimal length was set to 300 bp and primers were selected with the maximum number of CpG sites. Illumina tags were added to the 5′ end of each primer to enable recognition for sequencing (Illumina forward tag: 5′ACGACGCTCTTCCGATCT 3′; Illumina reverse tag: 5′ CGTGTGCTCTTCCGATCT 3′). Samples from placenta (first trimester and term), somatic tissue (blood and melanocyte), and melanoma cell lines were selected for targeted deep bisulfite sequencing experiments (Supplementary Table S11). DNA was not available from hESCs for TDBS assays; therefore, methylation was not assessed using this tissue.

Reads were aligned to target regions using Biq_Analyzer. Alignments were then used to calculate methylation scores. Individual CpG and mean CpG methylation values were generated for each amplicon and each sample.

### 4.5. Statistical Analyses

Statistical analyses were performed as described previously [51]. To determine whether individual genes were differentially expressed between somatic and early developmental tissues (placenta or hESCs), a Mann–Whitney test was used for non-normally distributed data and a Welch’s T-test was used for normally distributed data. Normality of datasets was determined with a D’Agostino and Pearson test. To assess differential expression between more than two datasets that were not normally distributed, a Kruskal–Wallis test with Dunn’s multiple comparisons was performed (nonparametric). To determine differential expression between more than two datasets that were normally distributed, a Brown–Forsythe and Welch ANOVA test was performed. All statistical analyses were carried out in Graphpad Prism.

## Data Availability

Publicly available data were previously generated from melanoma cell lines and are located at https://www.ncbi.nlm.nih.gov/geo, accessed on 14 June 2026 (see GSE16404 and GSE153595). In addition, publicly available data from term placental samples as well as from five hESC samples were obtained from the sequence read archive (SRA-https://www.ncbi.nlm.nih.gov/sra, accessed on 14 June 2026) (see Supplementary Tables S3 and S4). Additional RNA-Sequencing data are available from the corresponding author upon reasonable request.

## Supplementary Materials

The following supporting information can be downloaded at: https://www.mdpi.com/article/10.3390/ijms27135827/s1.
