# Supplementary material for: Transcriptional Activation of Transposable Element (TE)-Associated Genes Is Frequently Associated with Altered Promoter Methylation in Placenta and Melanoma
Source: Int J Mol Sci. 2026 Jun 27;27(13):5827. doi: 10.3390/ijms27135827 (PMC13361550; doi:10.3390/ijms27135827)
Supplement: Supplementary file 1 [file ijms-27-05827-s001.zip › ijms-4349266-supplementary.pdf]

Supplementary Tables:

Table S1. First trimester placental samples used for generation of RNA-Sequencing data.

Sample ID	Gestation	Cohort	RIN	Depth	Stranded	Ribo-depleted	Reads
F125	5.3	Otago	6.9	50M	No	Yes	150bp
F131	7	Otago	6.0	50M	No	Yes	150bp
F132	8.2	Otago	5.6	50M	No	Yes	150bp
A	7	Auckland	8.4	66M	Yes	Yes	125BP
C	11.1	Auckland	5.4	66M	Yes	Yes	125BP
E	8.6	Auckland	5	66M	Yes	Yes	125BP
G	11	Auckland	5.1	66M	Yes	Yes	125BP
I	9.2	Auckland	6.1	66M	Yes	Yes	125BP
J	12.5	Auckland	7.2	66M	Yes	Yes	125BP
K	9.1	Auckland	5.4	66M	Yes	Yes	125BP
L	7.5	Auckland	5.1	66M	Yes	Yes	125BP
M	10.6	Auckland	8.1	66M	Yes	Yes	125BP
O	11.1	Auckland	8.2	66M	Yes	Yes	125BP
P	12	Auckland	6	66M	Yes	Yes	125BP
Q	10.4	Auckland	6.4	66M	Yes	Yes	125BP
S	8.5	Auckland	5.4	66M	Yes	Yes	125BP
T	8.6	Auckland	5.2	66M	Yes	Yes	125BP

Table S2. Term placental dataset used for RNA-Sequencing analysis (Geastation in weeks; Delivery, OV – operative vaginal, UV – unassisted vaginal, CS – caesarean section in labour).

Sample ID	Gestation	Delivery	Depth	Stranded	Ribo	Reads
SRR3111523	40.4	OV	83 M	Yes	Yes	100PE
SRR3111524	40.9	UV	83 M	Yes	Yes	100PE
SRR3111525	39.3	UV	83 M	Yes	Yes	100PE
SRR3111526	41.1	UV	83 M	Yes	Yes	100PE
SRR3111527	41.6	UV	83 M	Yes	Yes	100PE
SRR3111528	41.4	UV	83 M	Yes	Yes	100PE
SRR3111529	41.3	UV	83 M	Yes	Yes	100PE
SRR3111530	41.1	OV	83 M	Yes	Yes	100PE
SRR3111531	41.4	CS	83 M	Yes	Yes	100PE
SRR3111532	41.4	CS	83 M	Yes	Yes	100PE
SRR3111533	40.7	CS	83 M	Yes	Yes	100PE
SRR3111534	40.6	UV	83 M	Yes	Yes	100PE
SRR3111535	39.9	OV	83 M	Yes	Yes	100PE
SRR3111536	40.6	UV	83 M	Yes	Yes	100PE
SRR3111537	39.9	UV	83 M	Yes	Yes	100PE
SRR3111538	40.6	UV	83 M	Yes	Yes	100PE

Table S3. Human embryonic stem cell RNA-sequencing datasets included in analysis. UK – unknown data.

Sample ID	Source	Depth	Stranded	Ribodepletion	Read length
hESC H9	Australian Biosearch	66M	Yes	Yes	125PE
hESC H7	Encode	UK	Yes	Yes	101PE
hESC H7	Encode	UK	Yes	Yes	101PE
SRR7643154	SRA	90M	Yes	Yes	100PE
SRR7643155	SRA	90M	Yes	Yes	100PE
SRR7643156	SRA	90M	Yes	Yes	100PE
SRR7643157	SRA	90M	Yes	Yes	100PE
SRR8949078	SRA	UK	No	Poly-(A)	100PE

Table S4. Healthy somatic control tissues used to generate RNA-Sequencing. UK – unknown data.

Tissue	Source	Donor	RIN	Depth	Stranded	Ribo	Reads
Brain	AMSBIO	24 M	8.6	66M	Yes	Yes	125PE
Heart	AMSBIO	29 M	7.8	66M	Yes	Yes	125PE
Liver	AMSBIO	64 M	8	66M	Yes	Yes	125PE
Kidney	AMSBIO	66 F	7.1	66M	Yes	Yes	125PE
Lung	AMSBIO	23 M	8	66M	Yes	Yes	125PE
Ovary	AMSBIO	52 F	7.4	66M	Yes	Yes	125PE
Testis	AMSBIO	24 M	7.9	66M	Yes	Yes	125PE
Melanocyte	Australian Biosearch	Cell line	9.3	66M	Yes	Yes	125PE
Melanocyte	UK	Cell	UK	UK	UK	Poly-	UK
Melanocyte	UK	Cell	UK	UK	UK	Poly-	UK

Table S5. Placental candidate genes selected for validation by RT-qPCR.

Gene name	Primer name	Primer Sequence (5'-3')	Product length	Exon
LINC00470	F	CCT ACA AAT TTT GGA CAT CCC A	146	1-2
	R	TGA TCA GGA GGG TGT GGT		
LINC00221	F	TTT ATG TGG TAC AGG GTT GGG	132	1-2
	R	TTG AAA GCC CAC AGC CT		
LINC01357*	F	GAT TCC AAT CTG GGC CTG ACA	138	1-2
	R	GGT TCT CCC ATC AGG ACC TC		
AP005262.2	F	ACTTCCACCAGCTAGCTCCA	207	1-2
	R	ATATTTTGGGAGGCCAAGGT		

Table S6. hESC candidate genes selected for validation by RT-qPCR.

Gene name	Primer name	Primer Sequence (5'-3')	Product length	Exon
LINC00698	F	GGC TCC AAC ATT CCC CAA A	115	1-2
	R	CAT GCA GTG TTT CTG ATG CTT		
LINC02575	F	GCC TCA TCT GGA ATC TCG GAA	301	Yes
	R	ATG ACA CTC TTC CCT TCC CCG		

Table S7. Samples used for RT-qPCR validation experiments.

Tissue	Samples	RNA-Seq	NanoString	TDBS
Placenta first	6	Yes	Yes	Yes
Placenta term	5	No	Yes	Yes
hESC	1	Yes	Yes	No
Blood	1	No	Yes	Yes
Melanocyte	2	No	Yes	Yes
Heart	1	Yes	Yes	No
Brain	1	Yes	Yes	No
Melanoma	6	Yes	Yes	Yes

Table S8. Placental candidates selected for TDBS assays.

Gene name	Primer name	Primer Sequence (5'-3')	Product length	CpGs
AP005262.2	F	TTTTATAGGTAAATGAAGTTTTTTTT	247	6
	R	AATAAAAACCATCCTAACTAACAC		
AC073264.3	F	TAGTTGAGTATTTAGGGTATTGGGG	185	6
	R	AATTTACAAATTCAAATAAAAAAAA		
LINC00470	F	TATTTTGGTTAATAGGGTGAAATTT	266	16
	R	TATTAAAATCATTAAACCTCATATTTTCTT		
PLAC4	F	TGGTAATTTTTGGGTGTTGTTATG	243	4
	R	AATTCCCCAATAAAAAATAAATCTCA		
HSD17B1	F	TGTTGTTTAGTTTGGAGTGTAATAG	153	5
	R	ATCTAACCAACATAAAAAAACCTC		
LINC01357*	F	TTGGAAGAGAAAAAGGAAAAGTTAT	216	14
	R	ATATCAAACCCAAATTAAAATCCAA		
LINC00221	F	GGATTATTTGTATTTTAGTTGTTTT	293	31
	R	ATCTCCAAACCCAACCCTATAC		

Table S9. hESC candidates selected for TDBS assays.

Gene name	Primer name	Primer Sequence (5'-3')	Product length	CpGs
LINC00698	F	TTTTGTTTTTGTTTGTTGTTATTTA	246	4
	R	CTACTACCCAATTCCAAAATTACTTC		
LINC02575	1-F	GGTAGAGGTAGAAATTGTTGGATGT	191	13
	1-R	ACTACTTAATAACCTAACTCTCCTCC		
	2-F	AGAGGTGTTATGTATGTTTTTATTT	213	13
	2-R	AAAAATTCCCTTAACACACTTAACC		
AC010624.5	1-F	GTTTTTTTAATTGTAAAGTGAGGAAAG	150	10
	1-R	CCTAAATCCCACCTAATATCAATAC		
	2-F	GAGTTTTTGTTAGTTTGTTTGGTT	208	29
	2-R	TTTCTAAAAAATATAATCCAACTAC		

Table S10. Samples selected for TDBS assays.

Tissue	Samples	Genes	Amplimers	RNA-Seq	qPCR
Placenta first	10	8	9	Yes	Yes
Placenta term	5	8	9	No	Yes
Blood	1	12	16	No	Yes
Melanocyte	1	12	16	No	Yes
Melanoma cell	10	12	16	Yes	Yes

Table S11. Differentially methylated CpGs in placental-enriched lncRNA TE-derived genes common between melanocyte and melanoma cell lines RRBS data.

Chr	Start	End	TE-derived gene	No. CpGs	No. DMCs (P<0.05, methdiff >=20%)
1	113392443	113420493	*LINC01357*	17	-
2	3606064	3612432	*RNASEH1-AS1*	7	1
3	127783621	127872757	*RUVBL1*	55	6
4	188916925	188926200	*ZFP42*	6	2
4	189012427	189030757	*TRIML2*	11	3
4	189080859	189082311	*LINC02434*	5	5
5	27217821	27497101	*PURPL*	5	1
5	156822542	157002783	*ADAM19*	65	16
7	152341861	152373226	*XRCC2*	1	-
13	111516334	111522598	*LINC00346*	5	-
14	106938441	106977000	*LINC00221*	16	6
15	74471807	74504608	*STRA6*	10	1
15	90118700	90174287	*TICRR*	30	-
16	19713259	19729557	*KNOP1*	20	-
16	88870197	88875666	*CDT1*	58	-
17	39958199	39968856	*P3H4*	37	4
17	40701232	40707231	*HSD17B1*	23	-
18	1254384	1408345	*LINC00470*	2	2
18	1509023	2049511	*AP005262.2*	18	4
19	43696854	43711451	*PSG4*	3	2
19	43715943	43773682	*PSG9*	2	2
19	50640116	50651487	*AC010624.5*	12	1
21	30565801	30660526	*LINC00189*	5	4

Supplementary Figures:

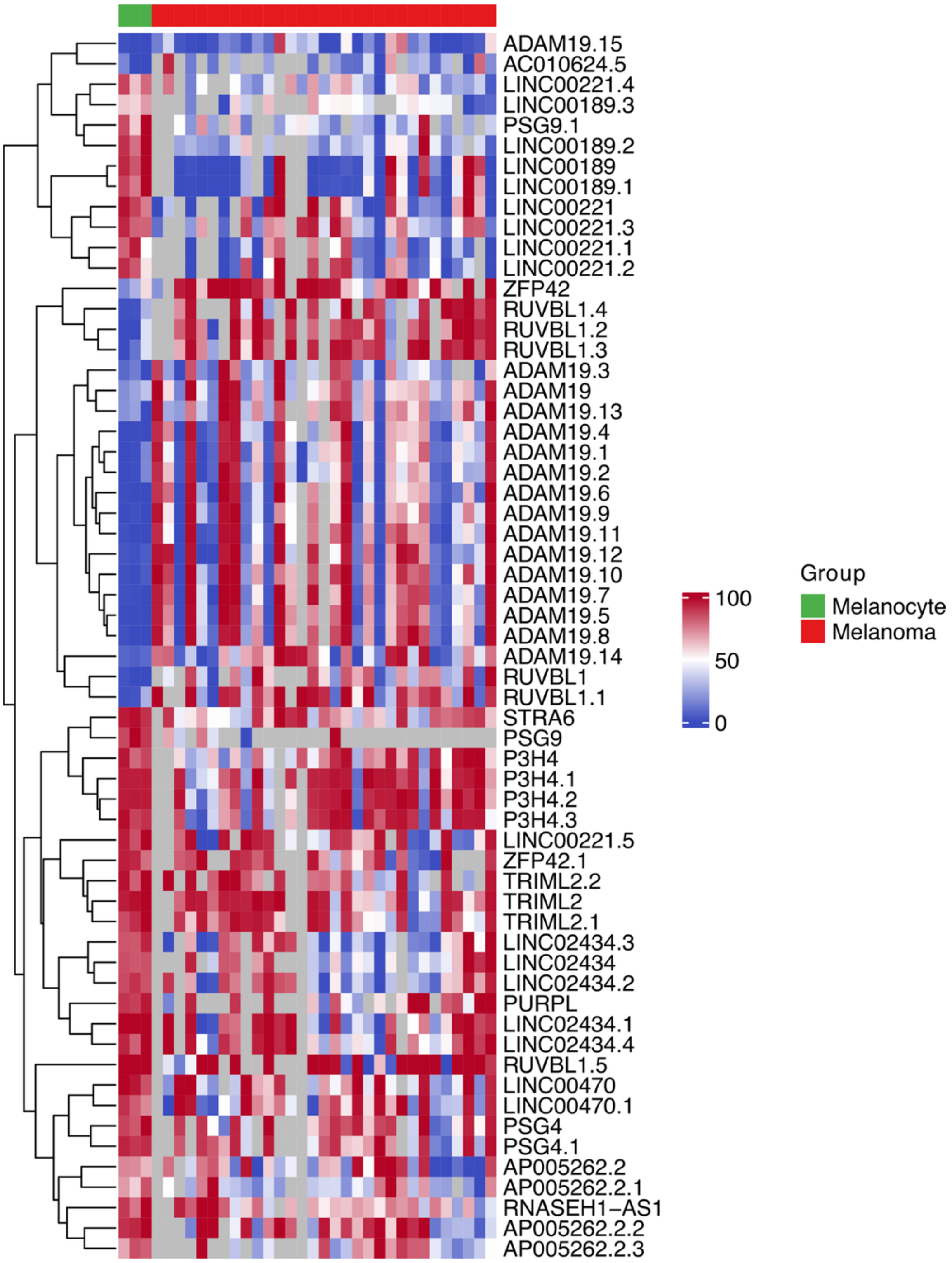

Figure S1. **Differential DNA methylation of placental-enriched lncRNA TE-derived genes between melanocyte and melanoma cell lines.** Heatmap of 60 differentially methylated CpGs (DMCs, $P < 0.05$, mean methylation difference ≥20%) from reduced representation bisulfite sequencing (RRBS) data of melanocytic (n=3) and melanoma cell lines (n=31) [1-5]; blue = unmethylated, red = fully methylated. Statistical analyses were performed independently for each genomic region using Welch's t-tests. See also Supplementary Table S11.

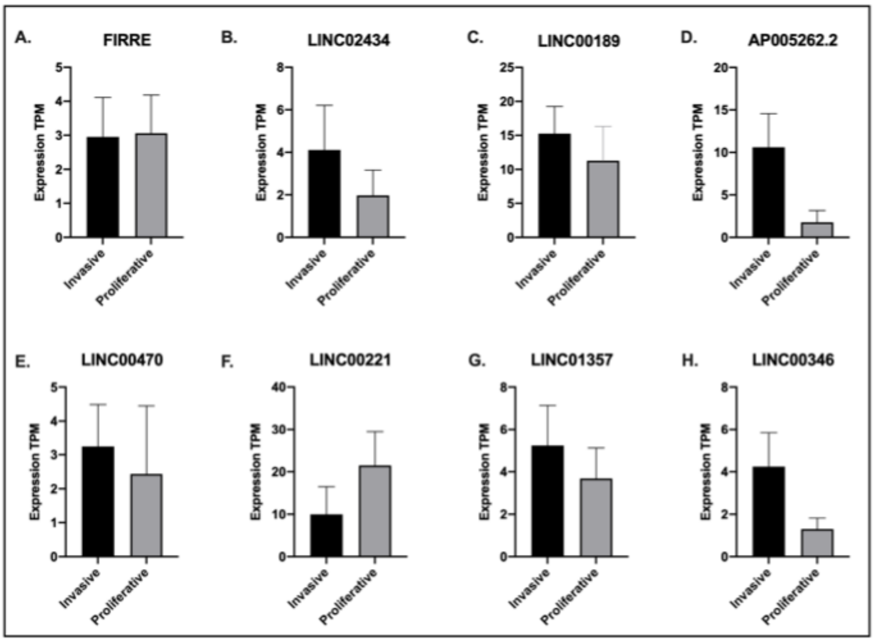

Figure S2. **Expression of placental-enriched lncRNA TE-derived genes in invasive and proliferative melanoma cell lines.** Invasive n = 6, proliferative n = 6. (“Invasive” is defined as the “Low MITF expression” signature melanoma subroup, while “Proliferative” is defined as the “High MITF expression” signature melanoma subgroup) [6]. No significiant differences were found between groups – Mann Whitney test.

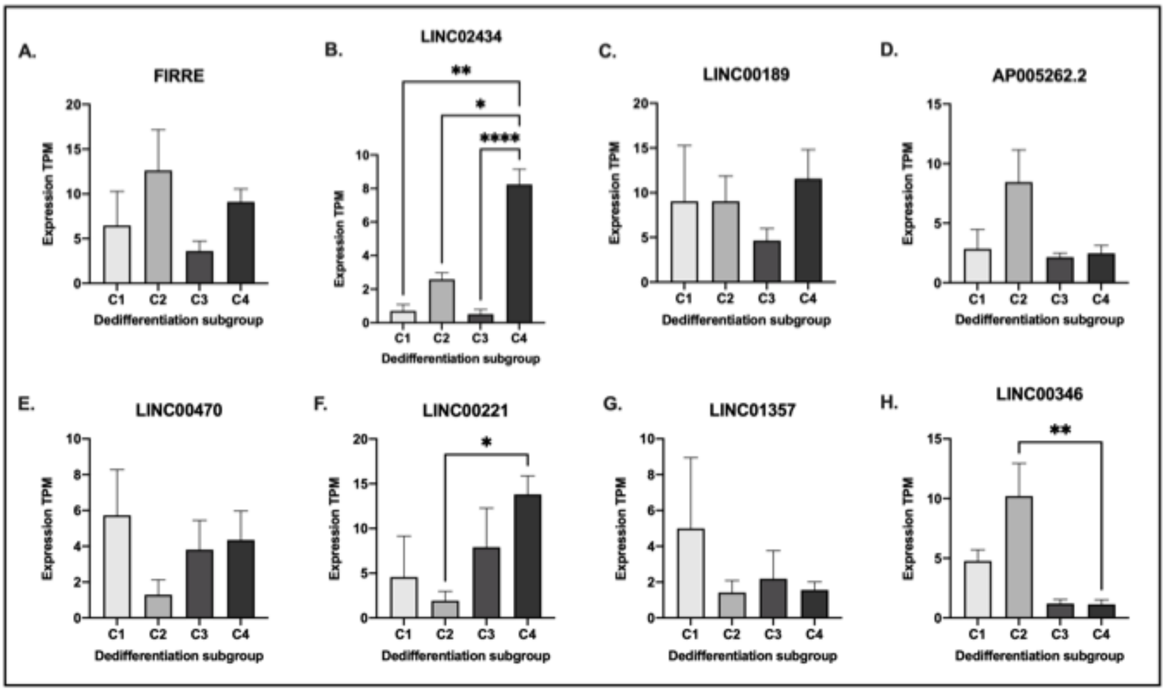

Figure S3. **Expression of placental-enriched lncRNA TE-derived genes in melanoma subgroups.** C1 n = 4; C2 n = 8; C3 n = 7; C4 n = 13 (C1 group = undifferentiated, C2 = neural crest like, C3 = transitory, C4 = melanocytic) [7]; **** = *p* value < 0.0001; *** = *p* value 0.0001 – 0.001; ** = *p* value < 0.001 – 0.01; * = *p* value < 0.01 – 0.05. Kruskall-Wallis test.

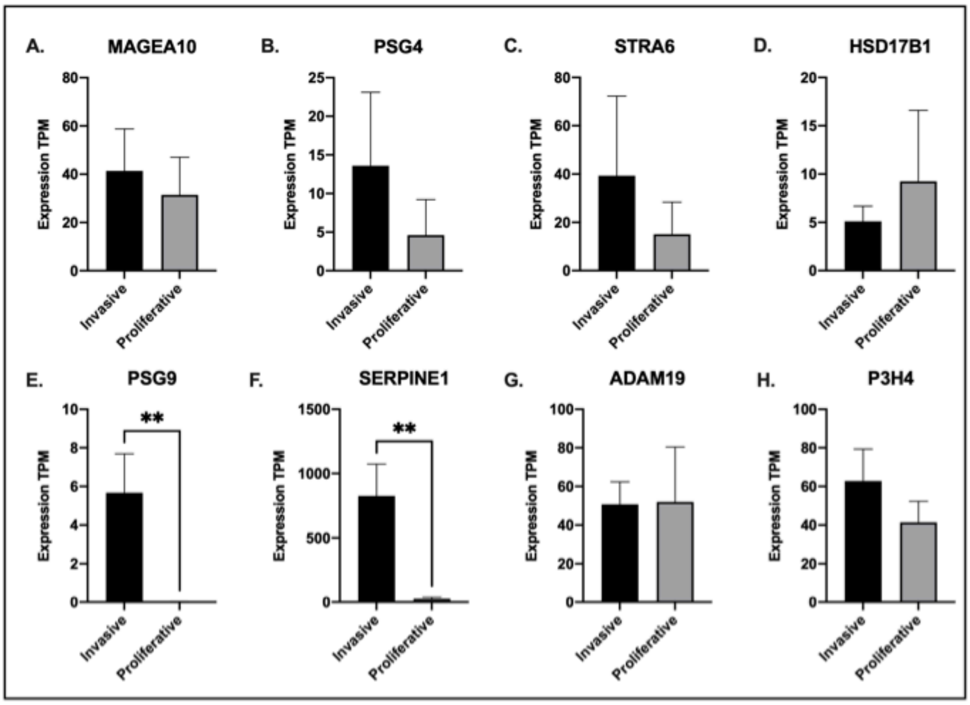

Figure S4. **Expression of placental-enriched protein-coding TE-derived genes in invasive and proliferative melanoma cell lines.** Invasive n = 6, proliferative n = 6 (See Supplementary Figure S2 legend for definitions). ** = *p* value 0.001 – 0.01 – Mann Whitney test.

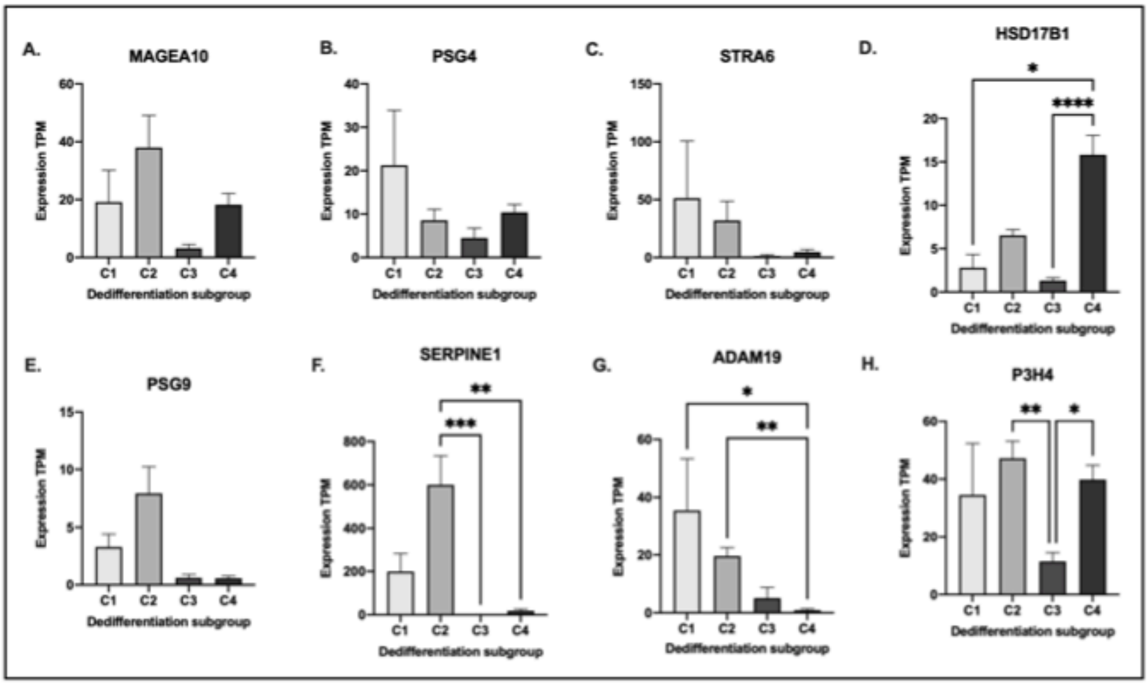

Figure S5. **Expression of placental-enriched protein-coding TE-derived genes in melanoma subgroups.** C1 n = 4; C2 n = 8; C3 n = 7; C4 n = 13 (See Supplementary Figure S3 legend for definitions); **** = *p* value < 0.0001; *** = *p* value 0.0001 – 0.001; ** = *p* value < 0.001 – 0.01; * = *p* value < 0.01 – 0.05. Kruskall-Wallis test.

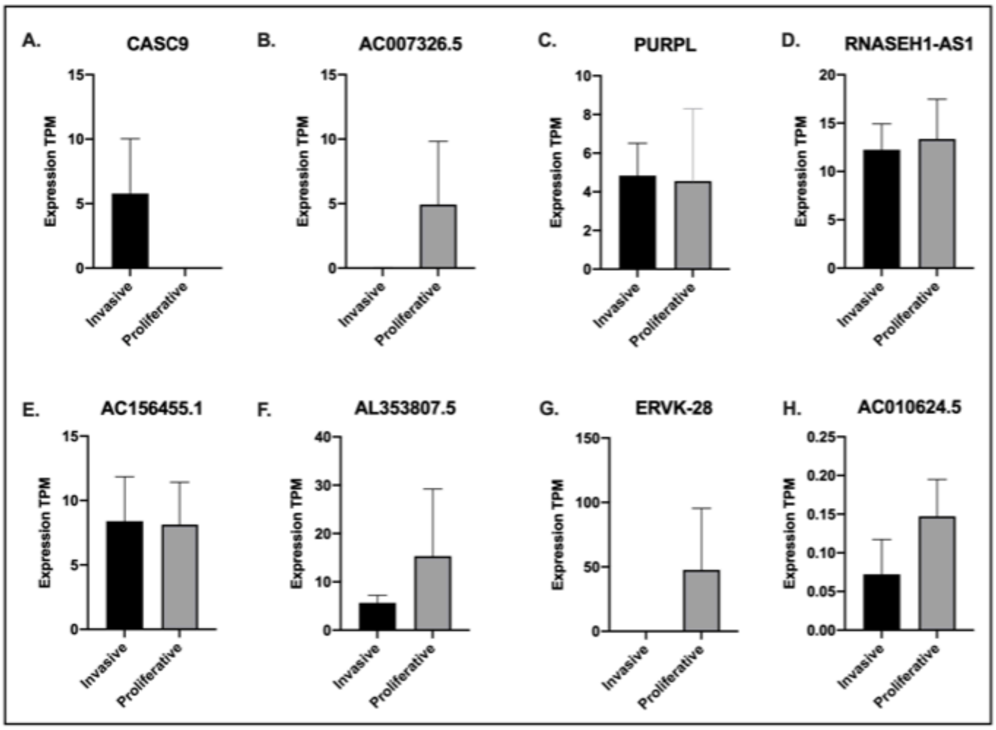

Figure S6. **Expression of hESC-enriched lncRNA TE-derived genes in invasive and proliferative melanoma cell lines.** Invasive n = 6, proliferative n = 6 (See Supplementary Figure S2 legend for definitions). No significiant differences were found between groups – Mann Whitney test.

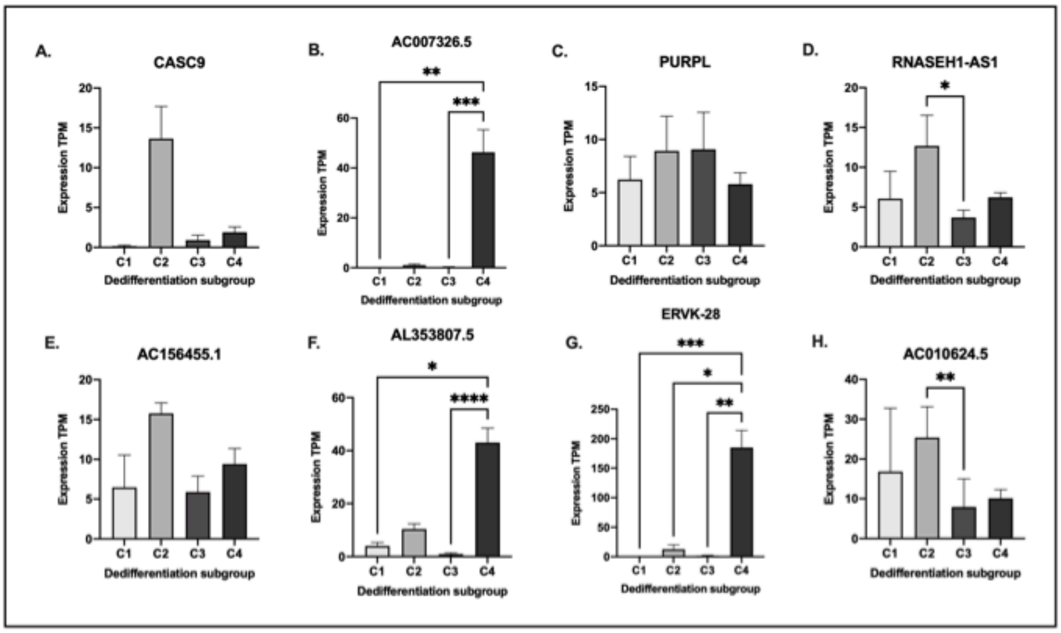

Figure S7. **Expression of hESC-enriched lncRNA TE-derived genes in melanoma subgroups.** C1 n = 4; C2 n = 8; C3 n = 7; C4 n = 13 (See Supplementary Figure S3 legend for definitions); **** = *p* value < 0.0001; *** = *p* value 0.0001 – 0.001; ** = *p* value < 0.001 – 0.01; * = *p* value < 0.01 – 0.05. Kruskall-Wallis test.

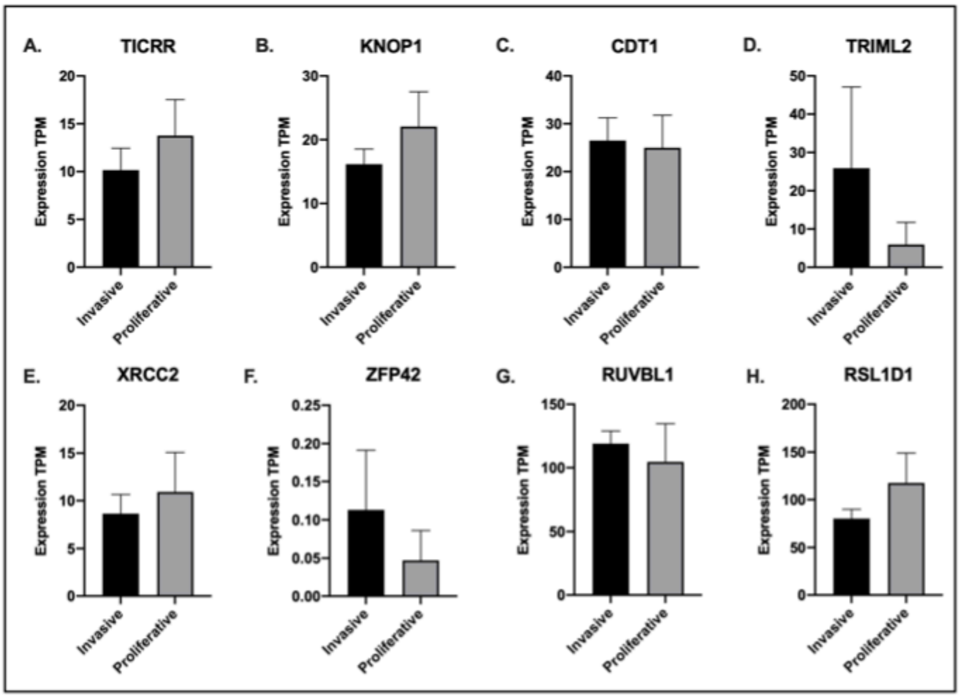

Figure S8. **Expression of hESC-enriched protein-coding TE-derived genes in invasive and proliferative melanoma cell lines.** Invasive n = 6, proliferative n = 6 (See Supplementary Figure S2 legend for definitions). No significiant differences were found between groups – Mann Whitney test.

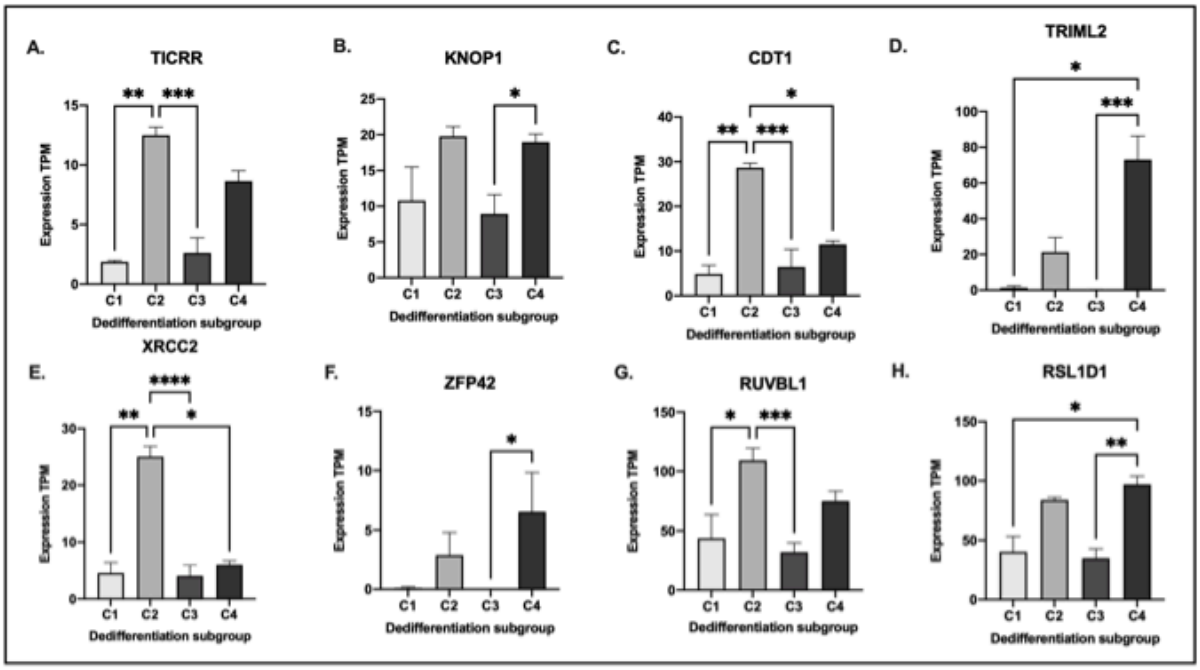

Figure S9. **Expression of hESC-enriched protein-coding TE-derived genes in melanoma subgroups.** C1 n = 4; C2 n = 8; C3 n = 7; C4 n = 13 (See Supplementary Figure S3 legend for definitions); **** = *p* value < 0.0001; *** = *p* value 0.0001 – 0.001; ** = *p* value < 0.001 – 0.01; * = *p* value < 0.01 – 0.05. Kruskall-Wallis test.

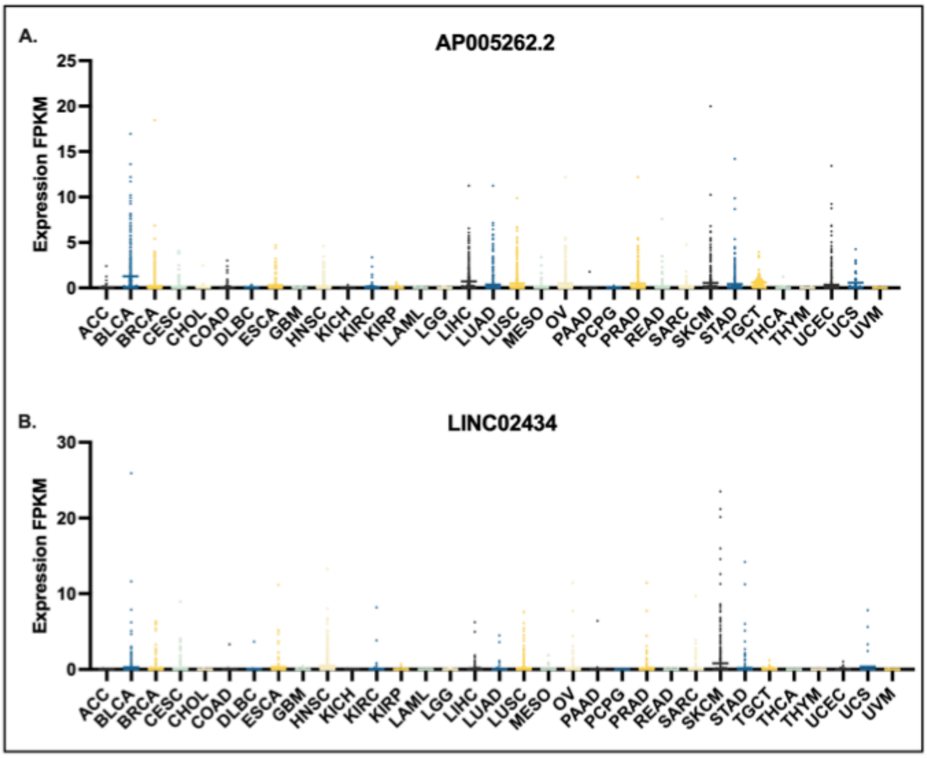

Figure S10. **Expression of two placental-enriched TE-derived genes in 33 different cancer types from TCGA.**

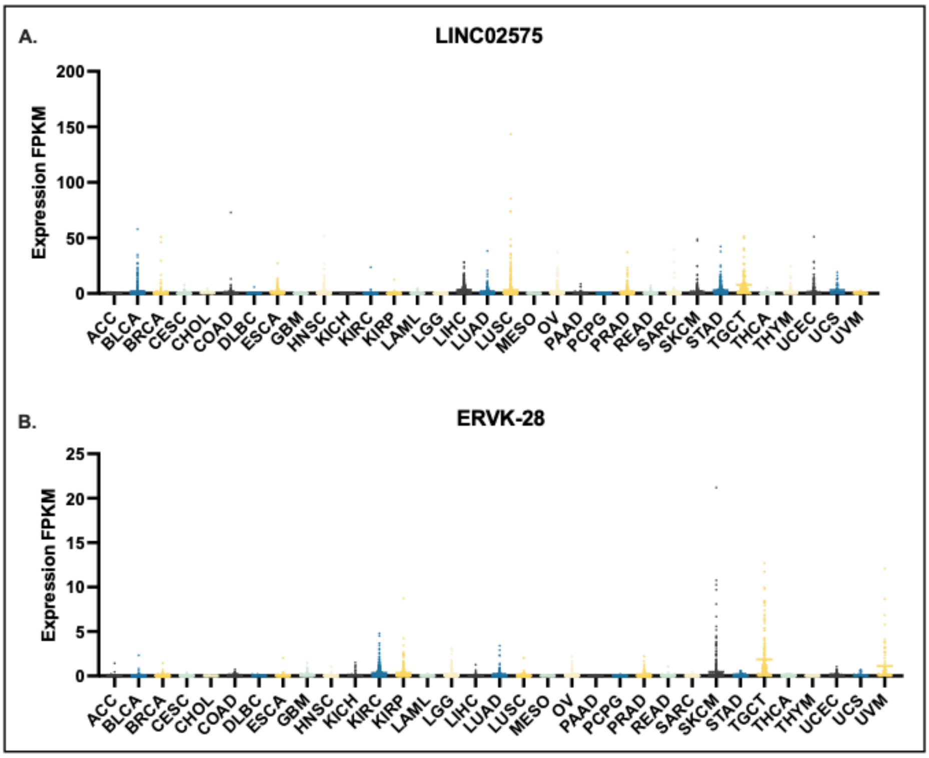

Figure S11. **Expression of two hESC-enriched TE-derived genes in 33 different cancer types from TCGA.**

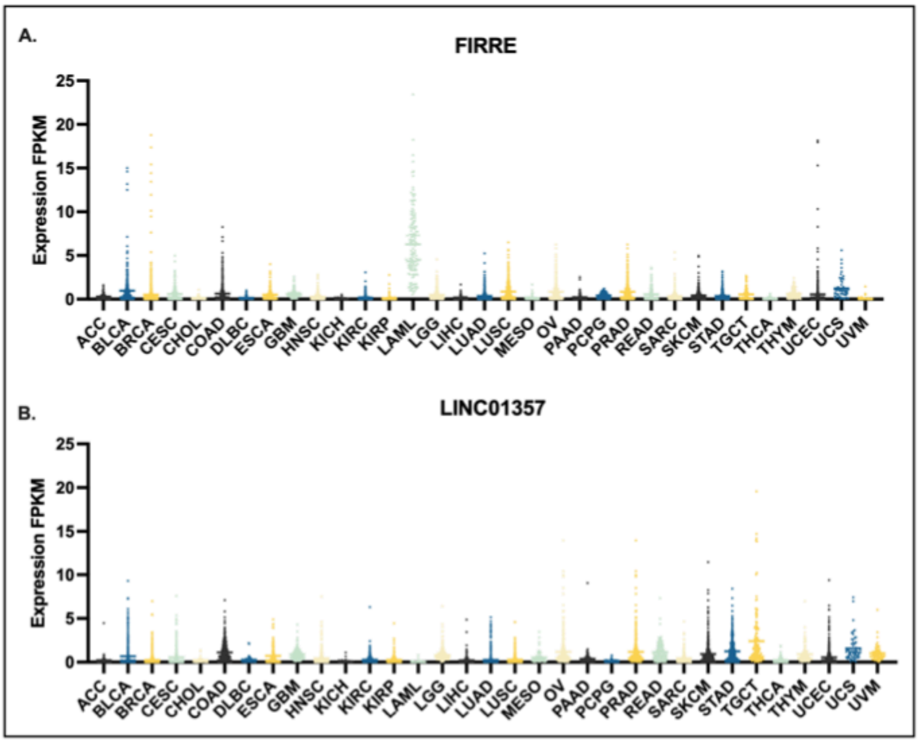

Figure S12. **Expression of two developmental-enriched TE-derived genes in 33 different cancer types from TCGA.**

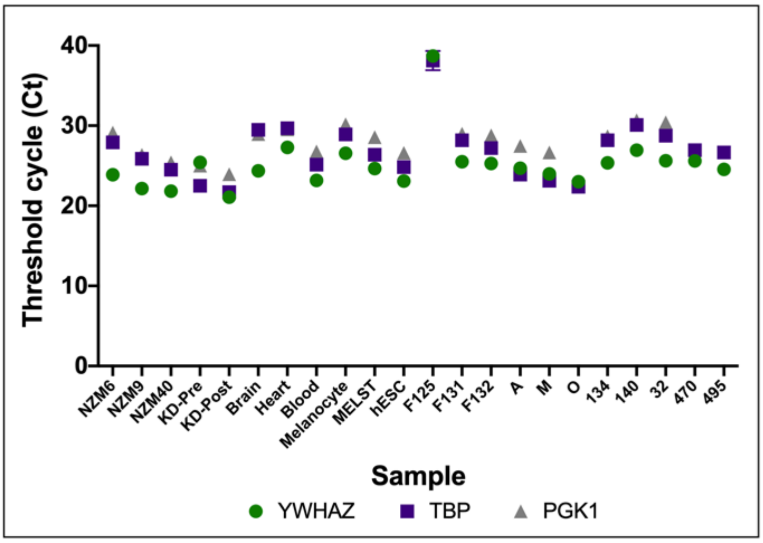

Figure S13. **Reference genes expression across different tissue and cell line RNAs used for RT-qPCR assays.** Ct scores are plotted on the Y-axis, and samples on the X-axis.

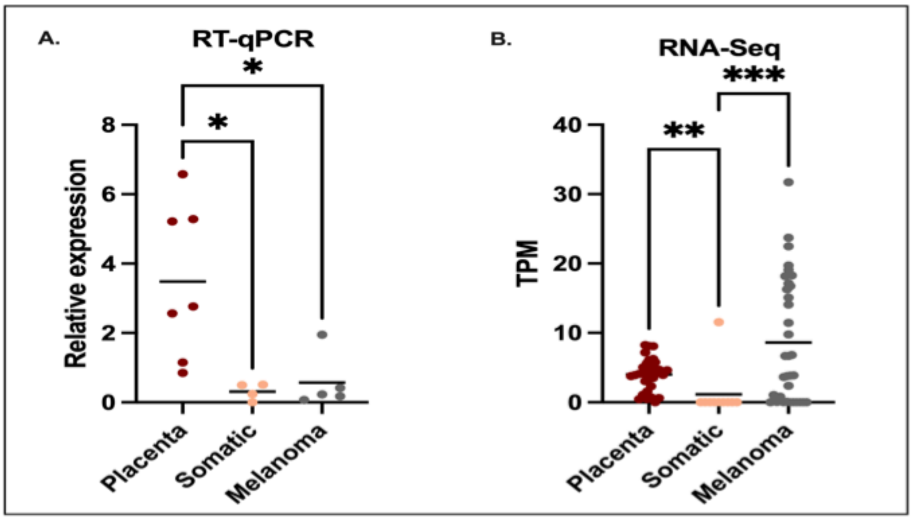

Figure S14. **Quantification of expression of *LINC00221* by RT-qPCR and RNA-Sequencing.** A. Expression of *LINC00221* in placental tissue, somatic tissue, and melanoma cell lines as quantified by RT-qPCR (placental tissues n=7, somatic tissues n=5, melanoma cell lines n=5) B. Expression of *LINC00221* in placental tissues, somatic tissues and melanoma cell lines. RNA-Seq datasets (placental tissues n=33, somatic tissues n=8, melanoma cell lines n=34). **** = *p* value < 0.0001, *** = *p* value 0.0001 – 0.001 ** = *p* value 0.001 – 0.01 * = *p* value 0.01 – 0.05. Kruskal-Wallis test.

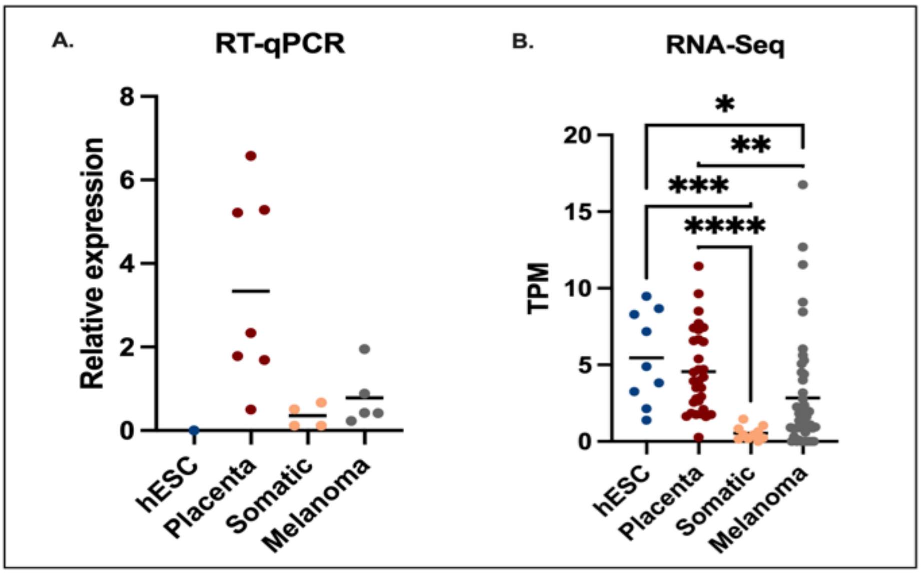

Figure S15. **Quantification of expression of *LINC01357* by RT-qPCR and RNA-Sequencing.** A. Expression of *LINC01357* in placental tissue, somatic tissue, and melanoma cell lines as quantified by RT-qPCR (placental tissues n=7, somatic tissues n=5, melanoma cell lines n=5) B. Expression of *LINC01357* in placental tissues, somatic tissues and melanoma cell lines. RNA-Seq datasets (placental tissues n=33, somatic tissues n=8, melanoma cell lines n=34). **** = *p* value < 0.0001, *** = *p* value 0.0001 – 0.001 ** = *p* value 0.001 – 0.01 * = *p* value 0.01 – 0.05. Kruskal-Wallis test.

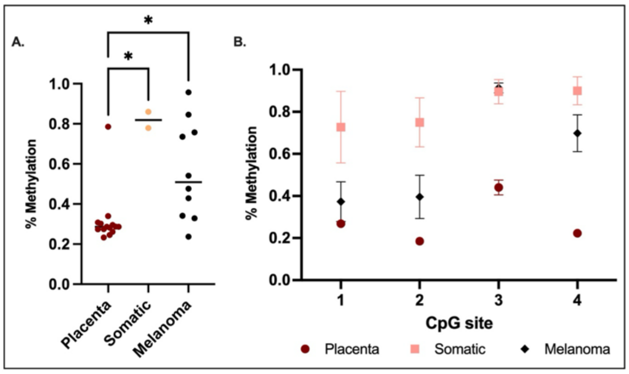

Figure S16. **DNA methylation analysis results for placental-enriched TE-derived gene, PLAC4**. A. Mean CpG methylation for the PLAC4 amplicon (*= P value = 0.01-0.04 – Kruskal-Wallis test) B. Methylation of each CpG within the PLAC4 amplicon. Placenta n=15, somatic n=2, melanoma cell line n=10.

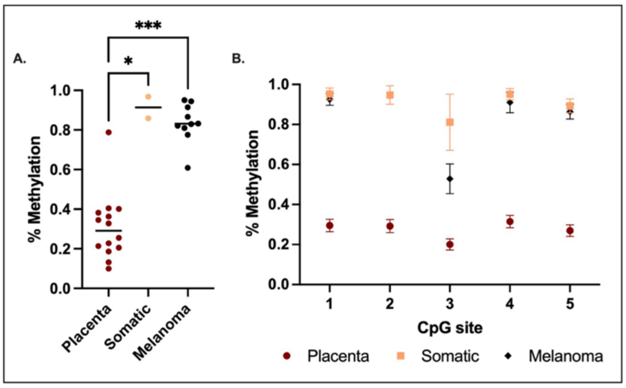

Figure S17. **DNA methylation analysis results for placental-enriched TE-derived gene, HSD17B1.** A. Mean CpG methylation for the HSD17B1 amplicon (*=P value=0.0183 ***=P value=0.0004 – Kruskal-Wallis test). B. Methylation of each CpG within the HSD17B1 amplicon. Placenta n=15, somatic n=2, melanoma cell line n=10.

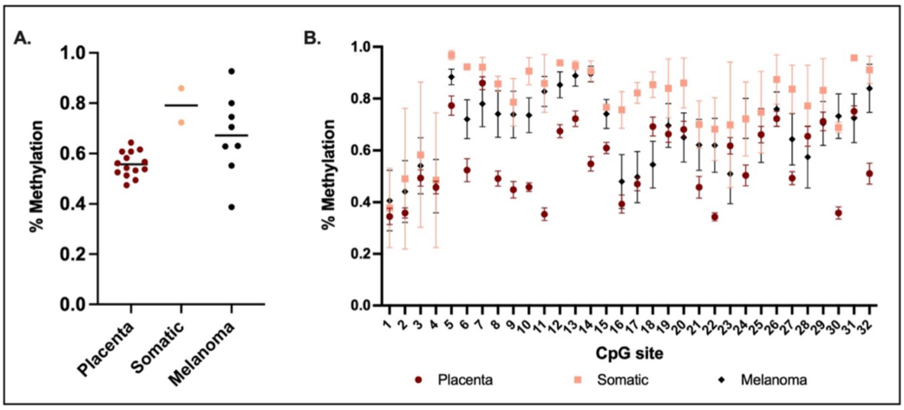

Figure S18. **DNA methylation analysis results for placental-enriched TE-derived gene, LINC00221**. A. Mean CpG methylation for the LINC00221 amplicon (Kruskal-Wallis test) B. Methylation of each CpG within the LINC00221 amplicon (Error bars represent SEM). Placenta n=15, somatic n=2, melanoma cell line n=10.

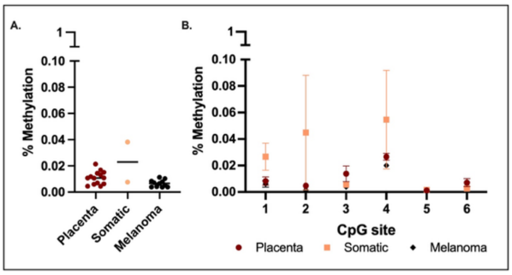

Figure S19. **DNA methylation analysis results for placental-enriched TE-derived gene, AC073264.** A. Mean CpG methylation for the AC073264 amplicon (Kruskal-Wallis test) B. Methylation of each CpG within the AC073264 amplicon (Error bars represent SEM). Placenta n=15, somatic n=2, melanoma cell line n=10.

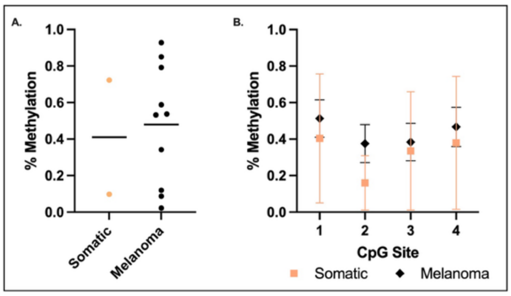

Figure S20. **DNA methylation analysis results for hESC-enriched TE-derived gene, LINC00698**. A. Amplicon location within the promoter region of LINC00698. B. Mean CpG methylation for the LINC00698 amplicon (P value = 0.9091 Mann-Whitney test) C. Methylation of each CpG within the LINC00698 amplicon (Error bars represent SEM). Somatic n=2, melanoma cell line n=10.

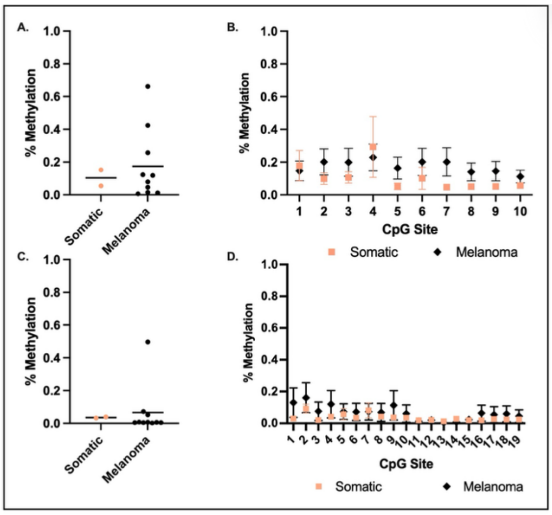

Figure S21. **DNA methylation analysis results for the hESC-enriched TE-derived gene, AC010624.5. A.** Mean CpG methylation for the AC010624.5 amplicon one (P value = 0.9091 Mann-Whitney test) B. Methylation of each CpG within the AC010624.5 amplicon one D. Mean CpG methylation for the AC010624.5 amplicon two (P value = 0.4848 Mann-Whitney test) E. Methylation of each CpG within the AC010624.5 amplicon tw0 (Error bars represent SEM). Somatic n=2, melanoma cell line n=10.

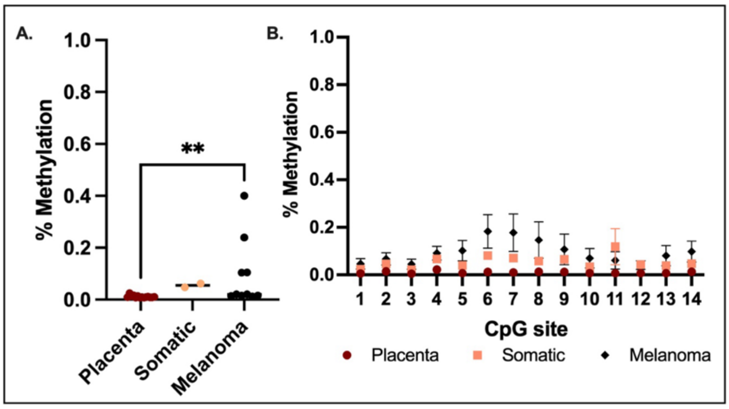

Figure S22. **DNA methylation analysis results for TE-derived gene, LINC01357**. A. Mean CpG methylation for the LINC01357 amplicon (**=P value = 0.0031 Kruskal-Wallis test) B. Methylation of each CpG within the LINC01357 amplicon (Error bars represent SEM). Placenta n=15, somatic n=2, melanoma cell line n=10.

References:

1. Rodger, E.J. *et al.* (2021) Comparison of Global DNA Methylation Patterns in Human Melanoma Tissues and Their Derivative Cell Lines. *Cancers (Basel)* 13. doi:10.3390/cancers13092123
2. Motwani, J. *et al.* (2021) Genome-wide DNA methylation and RNA expression differences correlate with invasiveness in melanoma cell lines. *Epigenomics* 13, 577-598. doi:10.2217/epi-2020-0440
3. Stockwell, P.A. *et al.* (2024) DMAP2: A Pipeline for Analysis of Whole-Genome-Scale DNA Methylation Sequencing Data. *Curr Protoc* 4, e70003. doi:10.1002/cpz1.70003
4. Chatterjee, A. *et al.* (2018) Marked Global DNA Hypomethylation Is Associated with Constitutive PD-L1 Expression in Melanoma. *iScience* 4, 312-325. doi:10.1016/j.isci.2018.05.021
5. Chatterjee, A. *et al.* (2017) Genome-wide methylation sequencing of paired primary and metastatic cell lines identifies common DNA methylation changes and a role for EBF3 as a candidate epigenetic driver of melanoma metastasis. *Oncotarget* 8, 6085-6101. doi:10.18632/oncotarget.14042
6. Jeffs, A.R. *et al.* (2009) A gene expression signature of invasive potential in metastatic melanoma cells. PLoS One 4(12):e8461. doi: 10.1371/journal.pone.000846
7. Tsoi, J. *et al.* (2018) Multi-stage Differentiation Defines Melanoma Subtypes with Differential Vulnerability to Drug-Induced Iron-Dependent Oxidative Stress. Cancer Cell. 33(5):890-904.e5. doi: 10.1016/j.ccell.2018.03.017
